# Burn Infections and Sepsis: Challenges and Future Prospects of Antibacterial Therapy

**DOI:** 10.3390/antibiotics15040383

**Published:** 2026-04-09

**Authors:** Ghazaleh Dadashizadeh, Margarita Elloso, Marc G. Jeschke

**Affiliations:** 1 Department of Biochemistry and and Biomedical Sciences, McMaster University, Hamilton, ON L8S 4L8, Canada; ghazaleh.dadashizadeh@taari.ca; 2 David Braley Research Institute, Hamilton, ON L8L 2X2, Canada; elloso@hhsc.ca; 3 Center for Burn Research, Hamilton Health Sciences, Hamilton, ON L8P 1A2, Canada; 4 Department of Surgery, McMaster University, Hamilton, ON L8S 4L8, Canada

**Keywords:** burn, sepsis, infection, antibiotics, resistance

## Abstract

Infectious complications remain a principal determinant of late morbidity and mortality following major thermal injury, reflecting a convergence of barrier disruption, microbial adaptation, and host immune dysfunction. The post-burn environment creates a uniquely permissive niche for pathogen persistence, characterized by altered tissue perfusion, biofilm formation, and dynamic shifts in microbial ecology toward multidrug-resistant organisms. Concurrently, profound and evolving changes in host immunity and metabolism reshape both susceptibility to infection and response to therapy. This review integrates current evidence across pathophysiology, microbiology, diagnostics, and treatment, with a focus on challenges that limit effective infection control in burn patients. Particular attention is given to diagnostic uncertainty arising from overlap between sterile inflammation and true infection, the clinical implications of biofilm-associated tolerance, and the impact of burn-specific pharmacokinetic variability on antimicrobial efficacy. We further examine emerging diagnostic and therapeutic innovations, including host-response profiling, rapid molecular detection platforms, and next-generation anti-infective strategies targeting microbial virulence, biofilm structure, and host immune pathways. Despite substantial scientific advances, translation into clinical practice remains constrained by limited burn-specific trials, heterogeneous definitions, and systemic barriers to antimicrobial development. Collectively, these challenges underscore the need for integrated, precision-based approaches that combine early source control, individualized antimicrobial optimization, and advanced diagnostic frameworks. Future progress will depend on coordinated efforts to standardize definitions, generate high-quality multicenter data, and align innovation with clinical applicability across diverse healthcare settings.

## 1. Introduction

Burn injuries remain a major global health burden, affecting millions of individuals annually and accounting for approximately 180,000 deaths worldwide, disproportionately impacting low- and middle-income countries [1,2,3]. In these settings, outcomes are often worsened by delayed access to surgical care, limited infection control infrastructure, and restricted availability of antimicrobial therapy and diagnostic resources.

Severe burns, typically involving ≥20% of total body surface area, are associated with a high risk of complications, particularly infection, sepsis, and multiple organ failure, with infectious complications representing a leading cause of late mortality [4].

The pathophysiology of burn injury creates a uniquely vulnerable host environment, characterized by loss of the protective skin barrier, exposure of large wound surfaces to microbial colonization, and profound systemic immune and metabolic dysregulation [5].

These alterations not only increase susceptibility to infection but also complicate clinical recognition, as burn-induced inflammation frequently mimics the physiological features of sepsis. At the same time, management is further challenged by evolving antimicrobial resistance, biofilm formation, and variability in antimicrobial pharmacokinetics [6].

Interpretation of the existing literature is limited by substantial heterogeneity across studies, including differences in diagnostic criteria, sampling methodologies, and institutional antimicrobial practices. As a result, reported rates of infection and resistance patterns vary widely and are not always generalizable across burn care systems.

Accordingly, this review integrates current evidence on the mechanisms, epidemiology, and management of burn-associated infections while critically examining ongoing controversies and key knowledge gaps that hinder the development of standardized, evidence-based clinical approaches.

**Box 1: Key controversies and evidence gaps in burn infection care**

Key unresolved issues include: (i) lack of standardized, burn-specific thresholds to distinguish colonization from invasive infection across sampling methods; (ii) competing sepsis criteria that yield different epidemiology and trial eligibility; (iii) uncertain clinical utility of biofilm diagnostics without validated management algorithms; (iv) limited burn-specific comparative trials for topical antimicrobial platforms and modern systemic agents; and (v) scarcity of multicenter prospective datasets linking PK/PD targets, biomarker trajectories, and clinical outcomes.

**Pathophysiology of Infection and Sepsis in Burns**

Severe burn injury induces a complex and dynamic systemic immune response initiated by the release of damage-associated molecular patterns (DAMPs), which activate innate immune pathways and drive widespread cytokine production [7,8]. This early hyperinflammatory phase is frequently followed by a state of immune suppression characterized by impaired neutrophil function, reduced antigen presentation, and lymphocyte dysfunction. Collectively, these alterations compromise pathogen clearance and increase susceptibility to infection, contributing to the high incidence of sepsis in patients with severe burns.

At the cellular level, burn-associated immune dysfunction includes reduced T-cell activity, decreased monocyte HLA-DR expression, and dysregulated neutrophil responses, reflecting a broad impairment of both innate and adaptive immunity [9].

Concurrently, full-thickness burns create a devascularized wound environment that supports microbial proliferation while limiting immune cell access and antimicrobial penetration [4]. This permissive niche facilitates progression from local colonization to invasive infection and systemic dissemination [10,11]. Clinical recognition of infection is further complicated by the overlap between burn-induced inflammation and sepsis, necessitating reliance on dynamic clinical indicators such as wound deterioration, unexplained physiological decline, or microbiological evidence of invasion [10]. In parallel, biofilm formation within burn wounds promotes microbial persistence and antimicrobial tolerance, contributing to recurrent or treatment-refractory infections [12].

Additional factors further amplify infection risk and sepsis severity during prolonged hospitalization, including secondary infections such as pneumonia and catheter-associated bloodstream infections [13].

Systemic physiological alterations, particularly capillary leak, microvascular dysfunction, and a sustained hypermetabolic state, impair tissue perfusion, drive catabolism, and exacerbate immune dysfunction [4]. Together, these processes accelerate progression to organ dysfunction, underscoring the distinct biological and clinical features of burn-associated sepsis [6,14].

Despite strong mechanistic insights, translation into clinically actionable strategies remains limited. Associations between specific immune alterations, such as monocyte HLA-DR suppression or lymphocyte dysfunction, and infection risk are inconsistent across studies, reflecting variability in burn severity, timing of assessment, and concurrent clinical interventions. As a result, prospective validation of immune-based biomarkers to guide antimicrobial decision-making in burn patients remains an important unmet need.

## 2. Epidemiology and Temporal Microbiology of Burn Wounds

### 2.1. Global Burden

Epidemiological data indicate that infectious complications occur in approximately 20–60% of hospitalized burn patients, representing a major contributor to morbidity during the acute and subacute phases of care [12,15]. The risk of infection increases proportionally with burn severity and extent of injury, reflecting greater disruption of the skin barrier, prolonged hospitalization, and increased exposure to invasive interventions. In patients with extensive burns, particularly those involving more than 40% of total body surface area, infection becomes nearly ubiquitous, with some cohorts reporting that the majority of patients develop positive wound and/or bloodstream cultures during their hospital course [8,16].

This high burden of infection underscores the central role of infectious complications in shaping clinical outcomes after severe burn injury and highlights the importance of early surveillance, prevention strategies, and targeted antimicrobial management.

### 2.2. Temporal Microbiology of Burn Wounds

Early microbial colonization of burn wounds is typically dominated by Gram-positive organisms, particularly *Staphylococcus aureus*, reflecting endogenous skin flora and early wound exposure. As hospitalization progresses, the microbial landscape shifts toward Gram-negative pathogens, including *Pseudomonas aeruginosa*, *Acinetobacter baumannii*, and members of the Enterobacteriaceae family, driven by hospital environmental exposure, invasive interventions, and antimicrobial selective pressure [17]. Additional Gram-negative organisms, such as *Klebsiella pneumoniae*, *Escherichia coli*, *Acinetobacter baumannii*, and *Enterobacter* species, are frequently isolated, particularly in patients with prolonged hospitalization or intensive care unit admission [18,19,20,21]. Among fungal pathogens, *Candida* species are most commonly identified, including *Candida albicans*, *Candida glabrata*, *Candida tropicalis*, *Candida parapsilosis*, and the emerging multidrug-resistant pathogen *Candida auris*. Filamentous fungi, such as *Aspergillus* and *Fusarium* species, may also cause invasive infections in severely immunocompromised burn patients [22,23,24,25,26]. This dynamic evolution of wound microbiology has important implications for empiric antimicrobial selection and highlights the need for continuous microbiological reassessment during hospitalization [27].

However, reported pathogen distributions and temporal trends are highly context dependent. Factors such as local microbial ecology (including unit design, water systems, staffing patterns, and outbreak history), sampling methodology (surface swab versus tissue biopsy), and institutional antimicrobial policies substantially influence observed microbiological profiles. Furthermore, many studies do not clearly distinguish between colonization and invasive infection, leading to overestimation of infection rates and limiting comparability across centers. These limitations underscore the need for standardized surveillance frameworks and harmonized diagnostic criteria in burn infection research.

## 3. Major Burn Wound Pathogens and Antimicrobial Resistance

The epidemiology of burn wound infections is increasingly defined by antimicrobial resistance, with multidrug-resistant organisms now representing a dominant component of the pathogen landscape in burn units [4]. This evolving resistance profile significantly constrains therapeutic options and complicates empiric antimicrobial selection, often necessitating broader-spectrum or combination regimens [21,28]. Table 1 summarizes the major burn-associated pathogens, their key resistance mechanisms, and commonly used antimicrobial options

### 3.1. Staphylococcus aureus

*Staphylococcus aureus* commonly colonizes burn wounds during the early post-injury period due to disruption of the skin barrier [4]. Methicillin-resistant *S. aureus* (MRSA) remains highly prevalent in burn centers worldwide and is a major cause of nosocomial infection [28]. In critically ill burn patients, MRSA infections may progress from local wound colonization to bloodstream infection or ventilator-associated pneumonia, contributing substantially to morbidity and mortality [17]. The high prevalence of MRSA has therefore strongly influenced empiric antimicrobial strategies in burn intensive care units [21,29].

### 3.2. Pseudomonas aeruginosa

*Pseudomonas aeruginosa* is a leading cause of late burn wound infections and is particularly challenging to treat due to its intrinsic resistance and ability to rapidly acquire additional resistance determinants [30,31,32]. Multidrug-resistant *P. aeruginosa* strains are increasingly reported in burn units and may exhibit resistance to carbapenems, fluoroquinolones, and aminoglycosides, sometimes leaving polymyxins or newer β-lactam/β-lactamase inhibitor combinations as the remaining therapeutic options [30,31,33,34].

### 3.3. Acinetobacter baumannii

*Acinetobacter baumannii* has emerged as an important nosocomial pathogen in burn centers, partly because of its remarkable ability to persist in hospital environments and spread during outbreaks [35]. Carbapenem-resistant *A. baumannii* is increasingly encountered in burn intensive care units and is associated with limited therapeutic options and increased mortality [5,35,36,37].

### 3.4. Enterobacteriaceae

Members of the Enterobacteriaceae family, including *Klebsiella pneumoniae*, *Escherichia coli*, and *Enterobacter* species, are frequently isolated from burn wounds and bloodstream infections in hospitalized patients. Resistance in these organisms is commonly mediated by β-lactamase enzymes, including extended-spectrum β-lactamases and carbapenemases, which significantly compromise the efficacy of many β-lactam antibiotics and complicate treatment strategies [38,39,40].

### 3.5. Fungi

Candida species represent the most common fungal pathogens, while molds such as *Aspergillus* and *Fusarium* may cause invasive infections in severely immunocompromised burn patients. Emerging pathogens such as *Candida auris* are of particular concern due to multidrug resistance and their capacity to cause hospital outbreaks [41,42,43,44].

### 3.6. Multidrug Resistance in Burn Infections

Multidrug-resistant (MDR) organisms have become a central challenge in the management of burn-associated infections. Pathogens such as methicillin-resistant *Staphylococcus aureus* (MRSA), multidrug-resistant *Pseudomonas aeruginosa*, *Acinetobacter baumannii*, and carbapenem-resistant Enterobacteriaceae are increasingly encountered in burn units and are associated with limited therapeutic options [45]. Infections caused by these organisms often necessitate the use of last-line agents, including polymyxins or advanced β-lactam/β-lactamase inhibitor combinations, highlighting the growing complexity of antimicrobial management in this population.

Recent advances have expanded the antimicrobial armamentarium against resistant Gram-negative pathogens, with agents such as ceftazidime–avibactam, ceftolozane–tazobactam, and cefiderocol demonstrating activity against otherwise difficult-to-treat infections [36,45,46,47,48,49,50]. However, burn-specific clinical outcome data remain limited, and the optimal role of these therapies, whether as first-line or salvage options, has yet to be clearly defined. Their use is further constrained by cost considerations, variable availability across healthcare systems, and the potential for resistance emergence during treatment.

Interpretation of resistance patterns in burn populations is additionally complicated by the predominance of data derived from outbreak-driven or intensive care–based cohorts, which may overestimate the baseline prevalence of resistance. Consequently, antimicrobial resistance trends should be contextualized within local epidemiology, institutional practices, and antimicrobial stewardship frameworks to guide appropriate therapeutic decision-making [28,51].

**Table 1:** Major burn wound pathogens, resistance mechanisms, and commonly used antimicrobial options.

Pathogen	Major Resistance Mechanisms	Antibiotics Commonly Resistant	Potential Active Agents
*Staphylococcus aureus* (MRSA) [52,53,54]	*mecA* (±*mecC*) → PBP2a (β-lactam resistance) [55] *erm* genes → MLSB resistance (inducible/constitutive) Reduced glycopeptide susceptibility (VISA; e.g., *vanA*-related)	Methicillin, oxacillin Most β-lactams (except anti-MRSA cephalosporins) Macrolides, clindamycin (iMLSB) Often fluoroquinolones	Vancomycin Linezolid Daptomycin Ceftaroline Tigecycline (salvage)
*Pseudomonas aeruginosa* [56,57,58,59]	Efflux pumps (Mex systems) Porin loss (OprD) AmpC/PDC β-lactamases Carbapenemases (e.g., VIM, IMP, NDM) Biofilm-associated tolerance	Many β-lactams (including carbapenems in resistant strains) Fluoroquinolones Aminoglycosides	Piperacillin–tazobactam (if susceptible) Ceftazidime, cefepime Ceftolozane–tazobactam Ceftazidime–avibactam Imipenem–cilastatin–relebactam Cefiderocol (non-UTI alternative) Polymyxins (salvage; colistin mainly for cystitis)
*Acinetobacter baumannii* [60,61,62,63]	OXA-type carbapenemases ADC cephalosporinases Efflux pumps and reduced permeability Biofilm formation Sulbactam resistance (β-lactamases + PBP mutations) [36,64]	Carbapenems Cephalosporins Fluoroquinolones Often aminoglycosides	Sulbactam–durlobactam + imipenem–cilastatin/meropenem (preferred) High-dose ampicillin–sulbactam + partner (alternative) Polymyxin B or colistin Minocycline or tigecycline (high-dose, combination) Cefiderocol (refractory/combination)
*Klebsiella pneumoniae* [65,66,67,68]	ESBL production Carbapenemases: -KPC -MBLs (NDM, VIM, IMP) -OXA-48-like Porin loss and efflux	Penicillins 3rd-generation cephalosporins Carbapenems (in CRE/CPE strains)	KPC: -Meropenem–vaborbactam -Ceftazidime–avibactam -Imipenem–relebactam OXA-48-like: MBL: -Ceftazidime–avibactam + aztreonam -Cefiderocol
*Escherichia coli* [38,69,70,71,72]	ESBL production (often inferred via ceftriaxone resistance) Plasmid-mediated AmpC ±Carbapenemases (in CRE strains)	Penicillins Cephalosporins Fluoroquinolones	Carbapenems (severe ESBL infections) TMP–SMX or nitrofurantoin (cystitis, if susceptible) Newer β-lactam/BLI (mainly for carbapenem-resistant isolates)
*Enterobacter* species [38,73,74,75,76]	Inducible chromosomal AmpC (derepression possible) ESBL production ±Carbapenemases	3rd-generation cephalosporins Penicillins	Cefepime (carbapenem-sparing option) Carbapenems Newer β-lactam/BLI (for resistant isolates)
Candida species [24,77,78,79]	Azole resistance: -*ERG11* mutations/overexpression -Efflux pumps Echinocandin resistance (FKS mutations) Biofilm-associated tolerance	Fluconazole and other azoles (species-dependent)	Echinocandins (first-line for invasive disease) Amphotericin B formulations Azoles (if susceptible; species/syndrome dependent)
*Candida auris* [26,80,81,82]	Multidrug resistance (MDR) High azole resistance Biofilm formation Emerging echinocandin resistance (*FKS1* mutations)	Azoles Amphotericin B (in some isolates)	Echinocandins (first-line) Amphotericin B (selected cases) Escalation per expert/CDC guidance (for echinocandin-resistant or pan-resistant strains)

## 4. Diagnostic Challenges and Emerging Diagnostics in Burn Infection

Accurate diagnosis of infection in burn patients remains a major clinical challenge. Severe burn injury induces a systemic inflammatory response that frequently fulfills systemic inflammatory response syndrome (SIRS) criteria in the absence of infection. As a result, commonly used clinical indicators of sepsis, such as fever, tachycardia, leukocytosis, and metabolic stress, lack specificity in this population and may reflect the underlying injury rather than true infection [2] (Table 2).

Microbiological cultures remain the cornerstone of diagnosis but are limited by both interpretive and temporal constraints. Superficial wound cultures often represent colonization rather than invasive infection, as burn wounds are rapidly populated by environmental and nosocomial organisms [5]. Quantitative tissue biopsy cultures exceeding 10^5^ CFU/g are considered the reference standard for invasive infection, yet their invasive nature limits routine use [6]. Blood cultures provide confirmation of systemic infection but require 48–72 h for pathogen identification and susceptibility testing, necessitating empiric therapy during early clinical decision-making [7].

The utility of laboratory biomarkers in distinguishing infection from sterile inflammation remains inconsistent. Conventional markers such as procalcitonin and C-reactive protein may support clinical assessment, but their interpretation is confounded by burn-induced inflammation. Similarly, cytokines such as interleukin-6 rise rapidly following injury irrespective of infection status, limiting diagnostic specificity. Emerging markers, including reduced monocyte HLA-DR expression and cytokine balance indices, show promise but remain investigational and lack validation for routine clinical use in burn populations [11,12].

A key limitation is that biomarker thresholds established in general critical care may not be directly applicable to burn patients, given the influence of burn size, timing, surgical interventions, transfusion, and hypermetabolic responses on baseline trajectories. Consequently, studies report variable performance for individual biomarkers, with limited external validation and inconsistent comparison against reference standards.

Clinical scoring systems developed for sepsis recognition in general critical care populations also demonstrate reduced reliability in burn patients. Tools such as the Sequential Organ Failure Assessment (SOFA) and quick SOFA (qSOFA) may misclassify patients due to overlapping physiological abnormalities inherent to burn injury [11]. Burn-specific sepsis criteria proposed by the American Burn Association improve contextual relevance but have not been universally adopted and may still involve trade-offs between sensitivity and specificity, leading to inconsistent classification across studies [13].

Advances in diagnostic technologies aim to address these limitations by improving both speed and accuracy. Rapid molecular platforms, including multiplex polymerase chain reaction assays, enable simultaneous detection of pathogens and resistance genes within hours [15]. Metagenomic next-generation sequencing offers culture-independent identification of bacterial, fungal, and viral pathogens, including organisms that are difficult to detect using conventional methods [23,25].

Parallel efforts have focused on host-response–based diagnostics. Multiplex cytokine profiling (e.g., IL-6, IL-8, IL-10) and multi-omics approaches, including transcriptomic, proteomic, and metabolomic analyses, have identified signatures associated with the transition from sterile inflammation to infection, suggesting potential for improved diagnostic discrimination [23,24].

Given the complexity of burn-induced immune dysregulation, it is unlikely that a single biomarker will reliably distinguish infection from sterile inflammation. Future diagnostic strategies will likely require integrated frameworks combining rapid pathogen detection, host-response profiling, and clinical decision-support systems. Emerging machine learning approaches that incorporate physiological data, laboratory trends, and molecular biomarkers may further enhance early recognition and risk stratification of burn-associated infection and sepsis [26] (Table 3).

Key Clinical Messages

- Distinguishing infection from sterile burn inflammation remains a major diagnostic challenge because systemic inflammatory responses are common after burn injury.
- Conventional microbiological cultures remain the diagnostic standard but may not reliably differentiate colonization from invasive infection.
- Biomarkers such as procalcitonin and C-reactive protein may assist in monitoring but lack specificity in burn patients.
- Emerging diagnostics, including rapid molecular testing, host-response biomarkers, and machine-learning approaches, may improve early infection detection in the future.

## 5. Pharmacokinetic/Pharmacodynamic Variability

Antimicrobial pharmacokinetics in burn patients differ substantially from those observed in other critically ill populations, reflecting dynamic and phase-dependent physiological alterations that complicate attainment of therapeutic drug exposure [4]. These changes frequently render standard dosing regimens inadequate, increasing the risk of subtherapeutic concentrations and treatment failure [94].

### 5.1. Early Phase (Resuscitation)

During the initial 48–72 h following burn injury, increased capillary permeability and aggressive fluid resuscitation drive significant intravascular-to-extravascular fluid shifts, resulting in tissue edema and expansion of the extracellular fluid compartment [4]. This leads to an increased volume of distribution for hydrophilic antibiotics, including β-lactams and aminoglycosides, thereby diluting plasma drug concentrations. Concurrently, transient reductions in cardiac output during burn shock may delay attainment of peak concentrations (C_max), while impaired renal and hepatic perfusion can alter drug clearance [95]. Together, these changes result in lower peak levels and prolonged distribution phases. Consistent with this variability, early post-burn vancomycin pharmacokinetics are highly unpredictable and influenced by both resuscitation intensity and individual patient factors.

### 5.2. Hypermetabolic Phase and Implications for Antimicrobial Dosing

As patients transition into the hypermetabolic phase, pharmacokinetic variability persists but is driven by different mechanisms. Augmented renal clearance is common and contributes to increased drug elimination, particularly for renally cleared agents such as β-lactams, aminoglycosides, and vancomycin [46,96]. Consequently, standard dosing regimens frequently result in subtherapeutic exposure due to shortened half-lives and increased clearance. To achieve adequate pharmacodynamic targets, dosing strategies often require intensification, including higher doses, increased dosing frequency, or prolonged and continuous infusion approaches [4]. Time-dependent antibiotics are commonly administered via extended or continuous infusion to optimize exposure, whereas concentration-dependent agents may require higher peak dosing. Observational pharmacokinetic studies, although limited in size, consistently support the need for dose adjustment in burn patients, including for aminoglycosides, β-lactams, daptomycin, and colistin [96,97]. In addition, burn-associated hypoalbuminemia increases the unbound fraction of highly protein-bound antimicrobials, further altering drug distribution and clearance, particularly in patients with burns exceeding 20% TBSA.

### 5.3. Therapeutic Drug Monitoring

Given the marked inter- and intra-patient variability, therapeutic drug monitoring (TDM) is essential for optimizing antimicrobial therapy in burn patients, particularly for agents with narrow therapeutic indices such as aminoglycosides and vancomycin [46,98]. Antibiotic dosing should be reassessed frequently to reflect evolving physiological changes, including early fluid shifts, hypermetabolism, and later organ dysfunction. Fixed dosing strategies are therefore often inadequate [99]. Individualized dosing guided by TDM, renal function, and clinical status, ideally in collaboration with clinical pharmacists, is critical to balance efficacy, toxicity, and resistance risk.

### 5.4. Limitations of Current Evidence

Despite consistent recognition of augmented renal clearance and expanded volume of distribution in burn patients, the supporting evidence is largely derived from small, heterogeneous pharmacokinetic studies. These studies often lack standardized sampling strategies and are rarely linked to clinically meaningful outcomes such as microbiological clearance, resistance emergence, or mortality. As a result, antimicrobial dosing in burn care remains guided primarily by physiological principles and therapeutic drug monitoring rather than robust burn-specific randomized trials, particularly for newer antimicrobial agents.

## 6. Biofilm Formation and Barriers to Antimicrobial Penetration

Burn wound biofilms are commonly formed by opportunistic nosocomial pathogens, including *Pseudomonas aeruginosa* and *Acinetobacter baumannii*, which colonize the wound environment during hospitalization [5,85,100]. Within these structured communities, bacteria coordinate behavior through quorum-sensing systems that regulate virulence factor expression, biofilm maturation, and persistence [101,102].

### 6.1. Impact on Antimicrobial Tolerance

**Figure 1:**
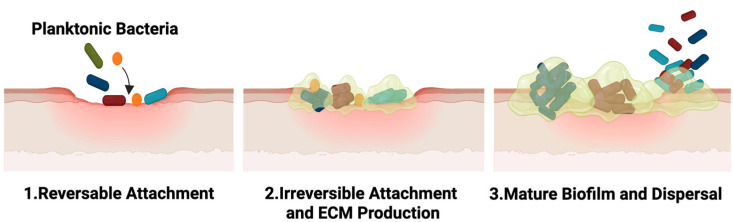
Stages of biofilm formation. Planktonic bacteria initially undergo reversible attachment to a surface, followed by irreversible adhesion accompanied by extracellular matrix (ECM) production. This leads to the development of a mature biofilm structure, from which bacterial cells can disperse to colonize new sites.

Biofilm architecture confers substantial antimicrobial tolerance through both physical and physiological mechanisms. The extracellular polymeric matrix limits antibiotic diffusion and establishes chemical gradients that reduce drug penetration into deeper biofilm layers [103,104]. Concurrently, biofilms harbor metabolically inactive subpopulations, including persister cells, which exhibit reduced susceptibility to antibiotics targeting actively dividing bacteria [5,105]. Together, these features promote persistent infection, delayed wound healing, and recurrent microbial colonization (Figure 1).

### 6.2. Diagnostic Challenges in Biofilm-Associated Infections

Detection of biofilm-associated infection remains challenging using conventional microbiological techniques. Standard culture methods primarily identify planktonic organisms and may underestimate both the presence and diversity of pathogens embedded within biofilms [27,106,107,108]. This limitation is further compounded by the polymicrobial nature of burn wound biofilms.

Advanced diagnostic approaches have improved biofilm detection in research and specialized settings. Microscopy-based techniques, including scanning electron microscopy and confocal laser scanning microscopy, enable visualization of biofilm structure and extracellular matrices within tissue samples [109,110]. Fluorescence imaging may assist in identifying areas of increased bacterial burden at the wound surface [109], while molecular methods such as PCR and metagenomic sequencing enhance detection of polymicrobial communities [93].

Despite these advances, routine clinical application remains limited by cost, technical complexity, and the absence of standardized diagnostic criteria. Consequently, biofilm involvement is often inferred clinically from indirect indicators such as delayed wound healing, persistent inflammation, recurrent infection, or inadequate response to antimicrobial therapy [99,103].

### 6.3. Anti-Biofilm Treatment Strategies

Effective management of biofilm-associated infections requires a multimodal approach. Mechanical or surgical debridement remains the cornerstone of therapy, disrupting the biofilm matrix, removing necrotic tissue, and enhancing penetration of systemic and topical antimicrobials [111,112]. However, optimal timing, frequency, and depth of debridement remain variable across centers, and burn-specific comparative outcome data are limited.

Adjunctive strategies aim to disrupt biofilm structure and improve antimicrobial access. Agents such as N-acetylcysteine can destabilize extracellular polymeric substances and have demonstrated antibiofilm activity in experimental and wound models, although clinical validation in burn populations remains limited [113,114]. Enzymatic debridement therapies show similar promise but lack robust randomized evidence [115,116,117]. Biological approaches, including larval therapy, may support biofilm disruption through proteolytic and antimicrobial effects, though their use in modern burn care remains variable [118,119].

Topical therapies also contribute to biofilm management. Antiseptics such as octenidine and polyhexamethylene biguanide (PHMB) provide broad antimicrobial activity with favorable tissue compatibility, while advanced wound dressings incorporating silver or copper ions may reduce microbial burden in colonized wounds [120].

Overall, effective biofilm control in burn wounds requires integration of early wound excision, repeated debridement, optimized antimicrobial therapy, and advanced wound-care strategies aligned with contemporary clinical guidelines.

## 7. Topical Antimicrobial Strategies in Burn Care

Topical antimicrobial therapy remains a key adjunct in burn wound management, primarily aimed at controlling surface microbial burden while definitive surgical treatment and wound closure are achieved. Given the limited penetration of systemic antibiotics into avascular burn eschar, topical agents play a critical role in suppressing microbial proliferation and preventing progression from colonization to invasive infection. However, their role is supportive rather than definitive, as early excision and grafting remain the most effective strategies for infection control [121,122,123].

Topical antimicrobials achieve high local concentrations within the wound environment, enabling effective reduction in microbial burden with minimal systemic toxicity and reduced systemic selective pressure for resistance [124]. Accordingly, contemporary burn care integrates topical antimicrobial therapy with surgical management and advanced wound-care approaches.

Silver-based formulations remain among the most widely used topical agents due to their broad-spectrum activity against Gram-positive and Gram-negative bacteria as well as fungi. Their antimicrobial effects are mediated through disruption of cell membranes, inhibition of respiratory enzymes, and DNA damage [124,125,126]. While traditional agents such as silver sulfadiazine are effective, they may delay epithelialization and require frequent application. In contrast, modern silver-containing dressings provide sustained antimicrobial release, maintain a moist wound environment, and reduce dressing-change frequency, improving both patient comfort and care efficiency [27,125,126].

Newer antimicrobial dressings increasingly incorporate antiseptic agents such as PHMB and octenidine, which offer broad antimicrobial activity with relatively favorable tissue compatibility. Iodine-based formulations, including cadexomer iodine and povidone-iodine, are also used in selected settings due to their wide antimicrobial spectrum and activity against biofilm-associated organisms [5,121,127,128,129,130]. However, clinical effectiveness varies depending on burn depth, wound characteristics, and institutional protocols [131].

Mafenide acetate remains particularly useful for deep burns and poorly perfused tissues due to its ability to penetrate burn eschar and its activity against Gram-negative organisms, including *Pseudomonas aeruginosa* [122]. Its use, however, may be limited by adverse effects such as metabolic acidosis and local pain during application [5,123].

Despite these advantages, topical antimicrobial therapy has important limitations. High bacterial burden, devitalized tissue, and biofilm formation can restrict antimicrobial penetration and reduce efficacy [4,5,122,132]. In addition, prolonged or excessive use may impair wound healing through cytotoxic effects or contribute to the selection of resistant organisms. Consequently, optimal use of topical antimicrobials requires integration within a comprehensive burn care strategy that combines timely surgical source control, appropriate wound management, and antimicrobial stewardship [121].

## 8. Current Approaches and Best Practices

**Figure 2:**
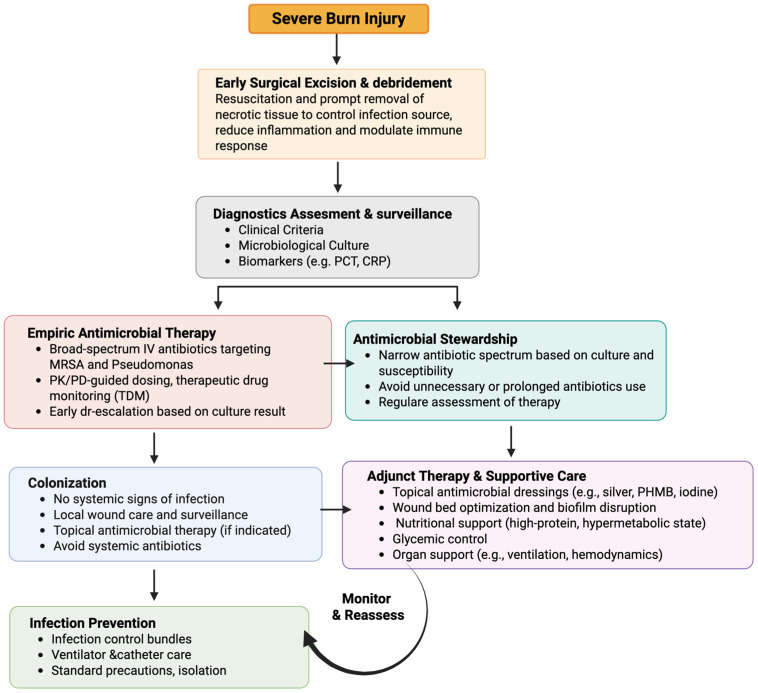
Integrated clinical framework for the diagnosis and management of burn wound infections and sepsis, highlighting early source control, diagnostic assessment, antimicrobial therapy, stewardship, adjunctive care, and continuous reassessment.

Despite the complexity of burn-associated infections and sepsis, clinical outcomes have improved through the adoption of integrated, evidence-informed management strategies. Contemporary burn care emphasizes early source control, individualized antimicrobial therapy, and rigorous infection prevention measures within a multidisciplinary framework. Collectively, these approaches have contributed to reductions in infectious complications and burn-related mortality. Key components of current practice are summarized below (Figure 2).

### 8.1. Early Excision and Grafting

Early surgical excision of necrotic tissue remains a cornerstone of infection prevention in major burns [122]. Typically performed within the first 48–72 h post-injury, early excision reduces bacterial burden, limits systemic inflammation, and restores barrier function through timely wound closure [4,47]. By eliminating devitalized tissue that serves as a reservoir for microbial proliferation, this approach provides effective surgical source control and is consistently associated with improved outcomes. In contrast, delayed debridement is linked to increased infection risk, sepsis, and adverse clinical trajectories [122].

### 8.2. Empiric Antibiotic Therapy Guided by Local Antibiograms

Prompt initiation of empiric systemic antibiotics is critical when infection or sepsis is suspected [123,133,134]. Empiric regimens should be guided by unit-specific microbiological surveillance and local antibiograms to reflect prevailing pathogens and resistance patterns [4,92]. Initial therapy typically provides coverage against both Gram-positive and Gram-negative organisms, including MRSA and *Pseudomonas aeruginosa*, often using combinations such as vancomycin with an antipseudomonal β-lactam [46,92,135].

Therapy should be reassessed early based on culture and susceptibility data, with de-escalation to targeted agents whenever feasible. This “start broad, then streamline” approach, combined with consideration of pharmacokinetic variability and clinical response, supports effective and individualized antimicrobial use [134,135,136].

### 8.3. Antimicrobial Stewardship and Avoidance of Unwarranted Use

Antimicrobial stewardship is integral to modern burn care, aiming to balance timely treatment with minimization of resistance and toxicity [134]. Structured stewardship strategies include avoidance of routine prophylactic antibiotics, daily reassessment of therapy, and pharmacokinetic-guided dose optimization, particularly for agents such as vancomycin and aminoglycosides, within a multidisciplinary framework [88].

A key principle is distinguishing colonization from true infection. In the absence of systemic signs, microbial isolation from wounds may be managed with local care and surveillance rather than prolonged systemic therapy [137]. Antifungal stewardship is also increasingly emphasized, as extensive antibacterial exposure predisposes patients to invasive fungal infections, necessitating risk-based approaches [42,97]. These strategies reduce unnecessary antimicrobial exposure without compromising clinical outcomes [137].

### 8.4. Infection Prevention Bundles

Infection prevention remains a central pillar of burn care. Bundled interventions include strict hand hygiene, appropriate use of personal protective equipment, dedicated equipment, and standardized environmental cleaning protocols [134]. Device-associated infections are mitigated through aseptic insertion techniques, routine reassessment, and implementation of ventilator and catheter care bundles [122,138,139,140].

Additional measures include contact isolation protocols and avoidance of communal hydrotherapy due to waterborne infection risk, with preference for individualized wound care systems [141,142]. Structural features such as single-patient rooms and HEPA-filtered environments may further support infection control, although the role of routine surveillance cultures varies across institutions [4,141].

### 8.5. Adjunctive Therapies

Adjunctive therapies may complement standard surgical and antimicrobial management in selected high-risk patients. Interventions such as intravenous immunoglobulin and granulocyte colony-stimulating factor have been explored in immunocompromised or critically ill burn patients; however, evidence remains limited, restricting their use to specific clinical scenarios [143,144].

Topical antimicrobial dressings containing silver, iodine, or chlorhexidine are widely used to control surface microbial burden between debridement procedures [128,145]. Negative-pressure wound therapy may further reduce bacterial colonization and improve graft adherence, particularly in large, well-prepared wounds [146].

### 8.6. Ongoing Controversies and Evidence Gaps

While several core practices, particularly early excision and source control, are supported by strong clinical rationale and consensus, other aspects of burn infection management remain less well defined. Optimal empiric antibiotic breadth, treatment duration, and the role of prophylactic systemic therapy continue to be debated. These uncertainties largely reflect the underrepresentation of burn patients in major sepsis trials and reliance on data extrapolated from heterogeneous critical care populations. Consequently, inter-center variability persists despite broadly shared stewardship principles.

Key Clinical Messages

- Early excision and wound closure remain the most effective strategies for preventing infection in severe burns.
- Empiric antibiotic therapy should be guided by local resistance patterns and rapidly de-escalated once microbiological data become available.
- Antimicrobial stewardship is essential to minimize resistance and avoid unnecessary antimicrobial exposure.
- Strict infection prevention practices and multidisciplinary care remain central to modern burn management.

## 9. Emerging and Future Therapeutic Strategies

Although conventional antimicrobial therapies remain central to burn infection management, their effectiveness is often limited by antimicrobial resistance, impaired drug penetration, and host immune dysfunction. Consequently, a range of innovative strategies is being explored to directly target resistant pathogens, disrupt biofilms, or augment host defense mechanisms.

### 9.1. Phage Therapy

Bacteriophage therapy has re-emerged as a promising approach for treating infections caused by multidrug-resistant bacteria [147]. Phages selectively infect and lyse bacterial cells, offering targeted antimicrobial activity while preserving the surrounding microbiome. Their ability to replicate at the site of infection may further enhance efficacy in environments with high bacterial burden, such as burn wounds [148].

Preclinical studies demonstrate activity against key burn-associated pathogens, including *Pseudomonas aeruginosa*, *Acinetobacter baumannii*, and *Staphylococcus aureus* [149,150]. However, widespread clinical implementation remains limited by regulatory challenges, the need for standardized production, potential development of phage resistance, and a lack of large-scale randomized trials in burn populations. Despite these barriers, phage therapy represents one of the most clinically advanced alternative antimicrobial strategies currently under investigation.

### 9.2. Enzybiotics

Enzybiotics are enzyme-based antimicrobials that target bacterial cell walls or disrupt biofilm matrices [151]. These include bacteriophage-derived endolysins and polysaccharide depolymerases, which can directly lyse bacteria or degrade extracellular polymeric substances that stabilize biofilms [152].

Given the central role of biofilms in chronic burn wound infections, enzybiotics offer a particularly attractive adjunctive strategy by enhancing antibiotic penetration and efficacy. However, current evidence is largely limited to experimental and early translational studies, and robust clinical validation in burn populations is lacking [100,153].

### 9.3. Antimicrobial Peptides

Antimicrobial peptides (AMPs) are key components of innate immunity with broad-spectrum activity against bacteria, fungi, and viruses. Their mechanisms include membrane disruption and interference with intracellular processes [154,155].

Beyond direct antimicrobial effects, many AMPs exhibit immunomodulatory properties that may promote wound healing and regulate inflammation [156,157]. This dual functionality makes them particularly attractive for burn care. Although several synthetic and engineered AMPs are under investigation, challenges related to stability, toxicity, and manufacturing costs continue to limit clinical translation [158,159,160].

### 9.4. Nanotechnology-Based Antimicrobial Delivery

Nanotechnology-based platforms offer novel approaches to improve antimicrobial delivery and therapeutic efficacy. Nanoparticle systems, including silver nanoparticles, liposomal formulations, and polymer-based carriers, can enhance drug penetration into infected tissues and biofilms while enabling controlled release [161,162,163].

Despite encouraging preclinical results, most nanotechnology-based strategies remain at early developmental stages. Key barriers include safety evaluation, regulatory approval, scalability, and demonstration of clear clinical benefit over existing therapies [164].

### 9.5. Anti-Virulence Therapies

Anti-virulence strategies aim to attenuate bacterial pathogenicity by targeting processes such as quorum sensing, toxin production, and adhesion, rather than directly inhibiting bacterial growth [165,166]. This approach may reduce selective pressure for resistance and preserve commensal microbial communities.

However, most anti-virulence therapies remain in early experimental stages, and their clinical effectiveness, particularly as monotherapy, is uncertain. These approaches are more likely to be effective when used in combination with conventional antimicrobials, especially in severe infections such as burn-associated sepsis [167,168].

### 9.6. Immunomodulatory Approaches

Burn injury induces profound immune dysregulation, characterized by an initial hyperinflammatory phase followed by sustained immune suppression, which increases susceptibility to infection and impairs pathogen clearance [13,169].

Therapeutic strategies aimed at restoring immune balance—including cytokine modulation, immune checkpoint targeting, and enhancement of innate immune responses—represent a promising avenue for improving infection outcomes. However, clinical evidence remains limited, and most approaches are still investigational [170].

### 9.7. Development of Novel Antibiotics

Despite advances in alternative therapies, the development of new antibiotics remains essential. Recent efforts have focused on agents with novel mechanisms of action that retain activity against multidrug-resistant pathogens. Innovations in high-throughput screening, structure-based design, and genomic discovery platforms have accelerated early-stage antibiotic development [171,172,173].

Nevertheless, translation into clinical practice remains constrained by economic barriers, regulatory complexity, and limited commercial incentives, contributing to a persistent gap between discovery and clinical availability.

### 9.8. Translational Outlook

Overall, most emerging anti-infective strategies in burn care remain supported by preclinical or early-phase evidence. Among these, bacteriophage therapy and antimicrobial peptides appear closest to clinical implementation, whereas nanotechnology-based systems and anti-virulence approaches are largely experimental.

Future progress will likely depend on integrated therapeutic strategies that combine biofilm disruption, targeted antimicrobial therapy, and immune modulation. Advancing these approaches will require well-designed, burn-specific clinical trials capable of demonstrating meaningful improvements in patient-centered outcomes.

## 10. Research Priorities and Implementation Needs

Sustained progress in the prevention and management of burn-associated infections and sepsis will require coordinated research efforts alongside effective implementation strategies. Multidisciplinary initiatives, including those led by the Society for Healthcare Epidemiology of America and the American Burn Association, have identified critical knowledge gaps and translational barriers that must be addressed to improve outcomes in burn populations [94]. Several priority areas have emerged to guide future research and clinical implementation.

### 10.1. Epidemiology and Surveillance

A central priority is the generation of high-quality epidemiologic data through standardized definitions and robust multicenter surveillance systems [174]. Current heterogeneity in diagnostic criteria for burn wound infection and burn-associated sepsis limits meaningful comparison across institutions and regions [175]. Establishing uniform case definitions and harmonized reporting standards would enable benchmarking, improve evaluation of interventions, and facilitate global comparisons [11,176,177].

Enhanced surveillance systems are also essential for tracking pathogen distribution, antimicrobial resistance trends, and clinical outcomes, thereby informing empiric therapy and infection prevention strategies at both local and global levels.

### 10.2. Diagnostics and Biomarkers

Earlier and more accurate detection of infection remains a major unmet need. Existing diagnostic approaches are limited by overlap between infection and burn-induced inflammation, underscoring the need for tools capable of distinguishing these states.

Promising directions include host-response biomarkers, multiplex cytokine profiling, and multi-omics approaches that capture dynamic immune signatures associated with infection and sepsis. Integration of biomarker data with clinical variables and predictive modeling may enable development of burn-specific risk stratification tools and clinical decision-support systems [175,176,177], supporting earlier diagnosis, more targeted antimicrobial use, and improved outcomes.

### 10.3. Antimicrobial Resistance and Therapeutic Strategies

Addressing antimicrobial resistance requires deeper understanding of burn wound microbiology, particularly the role of biofilms and polymicrobial communities in persistent infection and antimicrobial tolerance [178,179]. Characterizing temporal microbial dynamics during wound healing and treatment may reveal novel therapeutic targets and inform stewardship strategies [180].

In parallel, rigorous evaluation of emerging wound-care and antimicrobial approaches is needed. These include advanced dressings, topical antimicrobials, skin substitutes, and bioengineered tissues designed to limit microbial colonization [181,182]. Further research should also clarify optimal timing of wound closure and the role of adjunctive technologies, such as negative-pressure wound therapy and antimicrobial-impregnated dressings, within standardized care pathways [174,183,184].

### 10.4. Global Implementation and Burn Care Capacity

Translation of advances into clinical practice remains particularly challenging in low- and middle-income countries, where burn care infrastructure is often limited [3]. Structural constraints, including absence of dedicated burn units, overcrowding, limited isolation capacity, inadequate sanitation systems, and restricted access to microbiological diagnostics, contribute to high rates of nosocomial infection and antimicrobial resistance [173,185].

In these settings, scalable and cost-effective interventions offer the greatest impact. Early excision and timely wound closure remain critical but are often constrained by limited surgical capacity [186]. Strengthening referral systems and regional burn networks may improve access to definitive care.

Basic infection prevention measures, such as strict hand hygiene, standardized wound care protocols, and dedicated burn care teams, have demonstrated substantial impact even in resource-limited environments [46]. Cohorting strategies and minimization of unnecessary invasive devices may further reduce transmission of resistant organisms [187].

Given limited access to newer antimicrobial agents, clinicians often rely on older therapies, reinforcing the importance of locally adapted antimicrobial stewardship programs to preserve efficacy and limit resistance [188]. In parallel, investment in workforce training and education is essential. Capacity-building initiatives focused on resuscitation, surgical care, and infection control can significantly improve outcomes, even in the absence of advanced technologies [3].

International collaboration, including global burn registries and research networks, may further support data sharing, benchmarking, and dissemination of best practices. Ultimately, reducing infection-related mortality will require not only therapeutic innovation but also sustained investment in healthcare infrastructure and alignment of research priorities with real-world resource constraints.

## 11. Conclusions

Burn wound infections and sepsis remain major determinants of morbidity and mortality following severe thermal injury. The interplay of immune dysregulation, hypermetabolism, and loss of barrier integrity creates a uniquely permissive environment for infection while simultaneously complicating diagnosis and therapeutic management. These challenges are further amplified by the increasing prevalence of multidrug-resistant pathogens, biofilm-associated tolerance, and dynamic alterations in antimicrobial pharmacokinetics.

Contemporary burn care has improved outcomes through integrated strategies centered on early surgical source control, individualized antimicrobial therapy guided by local epidemiology, and rigorous infection prevention practices. However, persistent diagnostic uncertainty and evolving resistance patterns continue to limit optimal management, underscoring the need for more precise and context-specific approaches.

Importantly, the evidence base guiding burn infection management remains heterogeneous, with limited burn-specific randomized trials. As a result, many current practices rely on extrapolation from general critical care populations, observational studies, and expert consensus, contributing to ongoing variability in clinical practice.

Emerging advances, including rapid molecular diagnostics, host-response profiling, and novel antimicrobial strategies such as bacteriophage therapy, antimicrobial peptides, and nanotechnology-based delivery systems, offer promising opportunities to address these gaps. Future progress will depend on integrating these innovations into clinically applicable frameworks supported by well-designed burn-specific trials, standardized definitions, and multidisciplinary implementation strategies.

Ultimately, improving outcomes in burn-associated infections will require not only therapeutic innovation but also coordinated efforts to align research, clinical practice, and healthcare infrastructure across diverse care settings.

## Figures and Tables

**Table 2:** Key challenges distinguishing burn wound infection from burn-associated sepsis.

Domain	Burn Wound Infection	Burn-Associated Sepsis
Inflammatory baseline [6,10,27]	Local inflammation common in colonized wounds	Systemic inflammation persists independent of infection
Diagnostic specificity [83,84]	Surface cultures often reflect colonization	SIRS criteria overlap with sterile burn response
Microbial burden [5,85]	High local bioburden with biofilm formation	Hematogenous dissemination and secondary infections
Antimicrobial penetration [86,87]	Limited penetration into eschar and biofilm	Altered PK/PD reduces systemic drug exposure
Pathogen profile [10,27]	Early Gram-positive organisms progressing to Gram-negative bacteria and fungi	Predominantly MDR Gram-negative pathogens
Therapeutic strategy [88,89,90]	Topical agents and surgical debridement central	Early empiric systemic therapy with rapid de-escalation
Resistance pressure [85,91]	Repeated topical/systemic exposure selects MDR	Broad empiric therapy accelerates resistance

**Table 3:** Diagnostic Approaches for Burn-Associated Infection and Sepsis.

Diagnostic Method	Principle	Advantages	Limitations in Burn Patients
Wound surface culture	Microbial growth from wound swabs	Widely available; simple	Often reflects colonization rather than invasive infection
Quantitative tissue biopsy [10,12]	Microbial quantification (>10^5^ CFU/g)	Gold standard for invasive burn infection	Invasive; not routinely performed
Blood culture [92]	Detection of bloodstream pathogens	Confirms systemic infection	Slow turnaround (48–72 h)
Biomarkers (PCT, CRP) [84]	Systemic inflammatory markers	Useful for monitoring infection trends	Elevated after burn injury even without infection
Host immune markers (HLA-DR, cytokine panels) [9]	Immune dysfunction signatures	Potential to distinguish sterile inflammation vs. infection	Currently investigational
Molecular diagnostics (PCR panels) [27]	Rapid pathogen and resistance gene detection	Faster than culture	Cost and limited availability
Metagenomic sequencing [93]	Culture-independent pathogen detection	Detects polymicrobial infections	Expensive and not routine

## Data Availability

No new data were created or analyzed in this study. Data sharing is not applicable to this article.
